# Identification of potential causal variants for premature ovarian failure by whole exome sequencing

**DOI:** 10.1186/s12920-020-00813-x

**Published:** 2020-10-27

**Authors:** Haengun Jin, JuWon Ahn, YoungJoon Park, JeongMin Sim, Han Sung Park, Chang Soo Ryu, Nam Keun Kim, KyuBum Kwack

**Affiliations:** Department of Biomedical Science, CHA University, Gyeonggi-do, 13488 Republic of Korea

**Keywords:** Premature ovarian failure, Whole exome sequencing, *MCM8*, *MCM9*, *HFM1*, Gene variants

## Abstract

**Background:**

Premature ovarian failure (POF) is a highly heterogeneous disorder that occurs in 1% of women of reproductive age. Very few causative genes and variants contributing to POF have been detected, and the disease remains incompletely understood. In this study, we used whole exome sequencing (WES) to identify potential causal variants leading to POF.

**Methods:**

WES was conducted to identify variants in 34 Korean patients with POF, alongside 10 normal controls. Detected variants were filtered using a range of characterized bioinformatics analyses, and the machine learning tools, CADD and VEST, were used to predict pathogenic variants that could cause disease. VarSome was used for a comprehensive interpretation of the variants. Potential causal variants finally screened by these analyses were confirmed using Sanger sequencing.

**Results:**

We identified nine potential causative variants in genes previously associated with POF in 8 of 34 (24%) Korean patients by WES variant analysis. These potentially pathogenic variants included mutations in the *MCM8*, *MCM9*, and *HFM1* genes, which are involved in homologous recombination, DNA repair, and meiosis, and are established as causing POF. Using a combination of CADD and VEST, 72 coding variants were also identified in 72 genes, including *ADAMTSL1* and *FER1L6*, which have plausible functional links to POF.

**Conclusions:**

WES is a useful tool to detect genetic variants that cause POF. Accumulation and systematic management of data from a number of WES studies in specialized groups of patients with POF (family data, severe case populations) are needed to better comprehend the genetic landscape underlying POF.

## Background

When women are around 50 years old, ovarian function generally decreases, secretion of the female hormone estrogen declines, and menopause occurs. Menopause increases the incidence of various diseases, such as hypertension, depression, and obesity [1], with consequences for wider society. Against this background, premature ovarian failure (POF) is receiving increasing attention. POF, sometimes known as premature ovarian insufficiency, refers to the loss of ovary function before 40 years-of-age. POF occurs in 1% of all women, with 0.1% experiencing POF before 30 years-of-age [2]. POF is defined as amenorrhea, with at least 4 months loss of ovarian function, before 40 years-of-age and levels of follicle stimulating hormone (FSH) > 40 IU/L and estradiol < 50 pg/mL (measured twice, at least a month apart, by blood test) [3, 4].

Possible causes of POF include chromosomal and genetic abnormalities, autoimmune disease, viral infectious diseases, and congenital uterine anomalies. Further, surgery due to endometriosis, ovarian cancer, and chemotherapy are also risk factors for POF. Chromosomal abnormalities are detected in 10–15% of patients with POF and other associated genetic factors are detected in 20–25% [5], while 68% of POF cases have an idiopathic character, with no apparent cause, making it difficult to identify the exact etiology.

In the last decade, variants that may be causative for POF have been discovered using various methods. Some were identified in candidate genes for POF with known roles in various relevant processes, such as genital development, DNA replication, meiosis, immune function, and metabolic function [6]. Next generation sequencing (NGS) is a powerful tool to generate accurate results by analysis of complex genetic systems. Whole exome sequencing (WES) is an NGS approach that can be applied for discovery of rare variants within protein encoding gene regions that can cause changes in protein structure [7–9]. There have been no comprehensive WES studies of patients with POF in Korea.

Recently, various methods integrating machine learning for predicting the deleteriousness of variants have been developed, based on advances in computing performance. There are three main categories of mutation risk prediction methods: (1) prediction by phylogeny and sequence structure, (2) predictions based on evolutionary conservation of sequences, and (3) machine learning-based prediction methods that integrate data from various sources [10]. In this study, two methods, Variant Effect Scoring Tool (VEST) [11] and Combined Annotation Dependent Depletion (CADD) [12], were used to predict pathogenic variants. Ranked CADD values have been widely applied to predict and classify the deleteriousness of variants in several diseases [13, 14]. VEST, which can measure the deleteriousness of sequence insertions and deletions, is also established as a tool for variant classification [15]. For interpreting deleteriousness of variants, VarSome is an impact analysis tool for human genetic variation, providing a powerful analytical resource and a repository for accumulated global knowledge of the genomics community [16].

In this study, we analyzed WES data from 34 Korean patients with POF and 10 normal controls to identify potential causal variants. Bioinformatics analysis was used to characterize protein coding sequence variants corresponding to 131 known POF-candidate genes selected from public databases. We also describe novel variants that have not previously been associated with POF.

## Method

### Subjects

Unrelated patients (n = 37) diagnosed with POF with amenorrhea for at least 6 months before the age of 40 years, and with FSH plasma levels > 40 IU/L, were included in this study. WES was performed on 37 patients with POF (P1–P37) reported in a previous study, as well as ten normal control samples collected from postmenopausal women ≥ 48 years old who had not had POF and had no known genetic disease. All patients with POF were of Korean ethnicity and recruited from Seoul CHA Hospital. This study was approved by the Institutional Review Boards at CHA University and the Institutional Review Boards of CHA Bundang Medical Center.

### Whole exome sequencing

Genomic DNA (gDNA) was isolated from patient peripheral blood using the high-salt buffer method. A minimum of 100 ng gDNA per sample was used to generate sequencing libraries. Genomic DNA was fragmented to 150–200 bp using an E220 Focused-ultrasonicator (Covaris, Woburn, MA, USA). After fragmentation, libraries were prepared using Agilent’s SureSelect XT Human All Exon V4 + UTRs Enrichment kit, according to the manufacturer’s recommendations (Agilent Technologies, Santa Clara, CA, USA). Paired-end sequencing (100 bp) was performed on the HiSeq2500 platform, according to the manufacturer’s instructions (Illumina Inc., San Diego, CA, USA). FastQC software (version 0.11.4) was used to assess read quality, and Trimmomatic (version 0.32) was used to remove low quality reads (base quality < 20). Sequence reads were aligned into human reference genome (hg19/GRCh37) using BWA-MEM (version 0.7.7) [17] and converted into BAM files using SAMtools (version 1.3) [18]. Sorting and indexing of BAM files was also conducted using SAMtools, and Picard (version 1.14) was used to remove duplicate reads. The mean aligned-read depth value on target regions (approximately 70 Mb) was approximately 75× (range, 50–70×) with more than 68% of target regions covered by at least 20×, and 57% covered by at least 30×. Single nucleotide variant (SNV) and insertion/deletion (InDel) variants were called using the GATK (version 4.0.7.0) multiple-sample analysis pipeline (HaplotypeCaller, CombineGVCFs) and functionally annotated using SnpEffect (version 4.2), as well as in-house scripts.

### Annotation and variants filtering

**Fig. 1:**
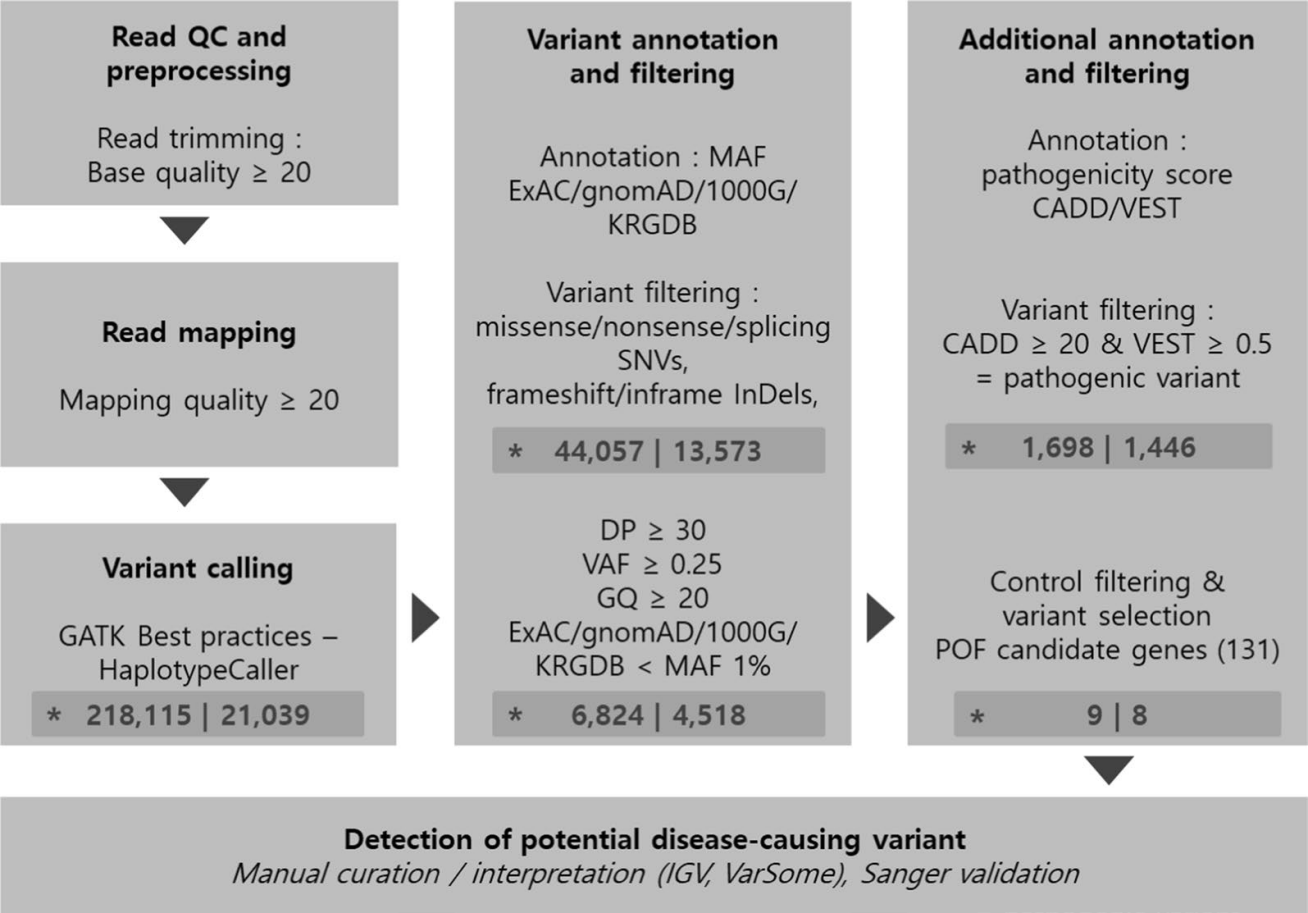
WES analysis pipeline. Variant filtering was performed at each stage of WES analysis. Variant counts and gene counts are shown at each stage (*). *DP* read depth, *VAF* variant allele frequency, *GQ* genotype quality

Preprocessing, variant calling, prediction of deleterious variants, and functional annotation were conducted to identify potential POF causative variants (Fig. 1). Only SNVs (non-synonymous, stop gain, stop loss, and splice sites) and InDel (frameshift, non-frameshift) variants that corresponded to coding regions and may affect protein function were collated. The obtained variants were filtered by location (target regions of the SureSelect XT Human All Exon V4 + UTR) and quality (read depth ≥ 20, variant allele frequency ≥ 0.25, genotype quality ≥ 20). Due to negative selection, deleterious variants are expected to have a lower frequency than neutral mutations. This theory has been proven in past large human genome studies [19]; therefore, variants were also filtered according to minor allele frequency (MAF) > 1% in the 1000 Genomes, Genome Aggregation Database (gnomAD), and Exome Aggregation Consortium (ExAC) databases [20, 21] to identify deleterious changes. In addition, variants in the Korean Reference Genome Database (KRGDB) [22] with MAF > 1% were filtered to remove common variants in the ethnic Korean population. Variants found in both control and patient samples were also filtered. Gene-disease association databases were used to create a list of genes associated with POF (Additional file 1: Table S1), and variants mapping to those genes were selected as potential variants causative of POF. Identified candidate variants were finally confirmed using the Integrative Genomics Viewer. In addition, the aggregated knowledge-based tool, VarSome, was used to comprehensively review variants [23].

### Pathogenicity prediction of variants

To reduce the range of candidate variants, variants were filtered using population allele frequency databases (ExAC, gnomAD, 1K genome, and KRGDB) and non-synonymous variants, predicted to result in protein modifications, were selected. To narrow the search even further and identify deleterious variants, additional annotation was conducted using the prediction tools CADD (version 1.4) and VEST (version 4). CADD is an ensemble method that integrates multiple annotations to predict the potential pathogenicity of variants, and is trained to identify potential pathogenic variants by integrating and combining several different annotation algorithms, based on a logistic regression model. VEST uses a training set, based on a random forest model, to distinguish pathogenic variants using databases of mutations classified as positive or negative. Both methods use machine learning-based algorithms and can be used to measure the deleteriousness of various types of short variants (missense SNV, nonsense SNV, splice site SNV, frameshift InDel, and non-frameshift InDel). A CADD (PHRED-like scaled) score ≥ 20 indicates that a substitution is predicted to be among the 1% most deleterious in the human genome. Therefore, CADD variants with scores ≥ 20 were classified as pathogenic variants and those with scores < 20 as benign. VEST scores were calculated using the CRAVAT web server (https://www.cravat.us). Variants with VEST scores ≥ 0.5 were classified as pathogenic and those with scores < 0.5 as benign. Finally, variants with both CADD scores ≥ 20 and VEST scores ≥ 0.5 were classified as potentially pathogenic candidate variants.

### Sanger validation

After filtering against multiple databases, candidate variants were re-sequenced using Sanger sequencing using an Applied Biosystems 3730xl DNA Analyzer. Targeted primers were designed using Primer3 software [24] (Additional file 1: Table S2). SeqMan 7.0 (DNAStar Lasergene, USA) was used for variant analysis of Sanger sequencing data.

## Result

To find potential causal variants of POF, we conducted WES using DNA from 37 patients diagnosed with POF and ten controls. Patients P1 and P31 were identified as XY females and data from patient P4 had biased GC content; therefore, data from 34 patients were included in our sequencing analysis [16]. Unfortunately, additional clinical information, such as XY karyotyping and ultrasound findings, were not available for these patients. Patient exomes were sequenced to an average sequencing depth of 100×. Clinical interpretation and evidence for the variants were carefully reviewed through VarSome.

### Identification of variants in genes previously associated with POF

Screening of genes known to cause POF (Additional file 1: Table S1) led to identification of nine plausible heterozygous variants (Table 1). These nine pathogenic variants in genes known to contribute to POF were carried by 8 of the 34 individuals (24%). Variants in *MCM8* (NM_001281521.1; c.839C>G; p.Ser280Cys, NM_001281521.1; c.1565C>T; p.Thr522Met) and *MCM9* (NM_017696.2; c.1330G>C; p.Val444Leu) were identified in 4 of 34 patients. A very rare variant (ExAC; MAF = 8.E−06) in *EIF2B3* (NM_020365.4; c.130G>A; p.Glu44Lys) was detected in P15, who also carried a variant in the *PREPL* gene (NM_001171603.1; c.1940G>A; p.Arg647Gln). P5 had an *ERCC6* variant (NM_000124.3; c.2510G>A; p.Arg837His) and an *HFM1* variant (NM_001017975.4; c.3047A>G; p.Gln1016Arg), which was also a very low frequency allele (ExAC; MAF = 8.E−06) found in P23. In addition, SALL4 (NM_020436.3; c.3149T>C; p.Ile1050Thr) and *TG* (P3; NM_003235.4; c.7364G>A; p.Arg2455His) variants were identified. The genes corresponding to the nine identified variants included participants in key biological processes, such as homologous recombination, DNA repair, and meiosis (*MCM8*, *MCM9*, and *HFM1*). The nine potential causal variants were confirmed by Sanger sequencing (Additional file 2: Figure S1).

**Table 1:** Potential causal variants detected in 8 patients with POF by whole-exome sequencing

GENE	Locus	dbSNP ID	Transcript ID	Sequence charge		Allele Frequency				Pathogenicity		
				cDNA	AA	ExAC	gnomAD	1000G	KRGDB	VEST	CADD	Patient ID
EIF2B3	chr1:45446711	rs201162647	NM_020365.4	c.130G>A	P.Glu44Lys	0.00001	0.00001	0.00020	0.00080	0.823	33.0	P15
ERCC6	chr10:50682161	rs77027474	NM_000124.3	c.2510G>A	P.Arg837His	0.00011	0.00007	0.00060	0.00500	0.822	32.0	P5
HFM1	chr1:91781465	rs755200362	NM_001017975.4	c.3047A>G	P.Gln1016Arg	0.00001	0.00001	.	0.00080	0.583	23.6	P23
MCM8	chr20:5943969	rs749490653	NM_001281521.1	c.839C>G	P.Ser280Cys	0.00022	0.00022	.	0.00273	0.616	22.9	P32
MCM8	chr20:5958571	rs201813827	NM_001281521.1	c.1565C>T	P.Thr522Met	0.00008	0.00010	0.00020	0.00409	0.612	27.5	P14, P28
MCM9	chr6:119150409	rs757364893	NM_017696.2	c.1330G>C	P.Val444Leu	.	0.00002	.	0.00136	0.630	25.9	P26
PRE PL	chr2:44549950	rs140355063	NM_001171603.1	c.1940G>A	P.Arg647Gln	0.00009	0.00009	.	0.00500	0.672	32.0	P15
SALL4	chr20:50400817	rs189552205	NM_020436.3	c.3149T>C	P.Ile1050Thr	0.00002	0.00002	0.00020	0.00136	0.978	25.1	P23
TG	chr8:134107412	rs2272707	NM_003235.4	c.7364G>A	P.Arg2455His	0.00070	0.00078	0.00120	0.00818	0.857	27.3	P3

VEST analysis generates values between 0 and 1, and scores ≥ 0.5 were classified as pathogenic variants and those < 0.5 as benign. CADD analysis generates a PHRED-like scaled value. Scores ≥ 20 were classified as pathogenic variants and those < 20 as benign. Variants satisfying both CADD ≥ 20 and VEST ≥ 0.5 were classified as pathogenic

### Identification of novel, candidate variants associated with POF

In addition to nine variants in genes associated with POF, 72 variants in 72 genes were found in two or more individuals in genes not previously associated with POF (Tables S3, S4). Of these, genes of particular interest, based on their known functions, included identical variants in two individuals found in *ADAMTSL1* and *FER1L6*, which are genes associated with the development of reproductive organs and folliculogenesis. The ADAMTS Like 1 (*ADAMTSL1*) variant (NM_001040272.5; c.397G>A; p.Glu133Lys) was found in two individuals. This variant generated high risk scores using both VETS (0.924) and CADD (32.0). The variant in *FER1L6* was c.814G>A; p.Gly272Ser (NM_001039112.2).

## Discussion

In this study, we recruited 34 Korean patients clinically diagnosed with POF and performed WES to identify pathogenic variants. Nine variants were detected that corresponded to genes known to potentially cause POF. Among these genes, Minichromosome maintenance 8 (*MCM8*) and Minichromosome maintenance 9 (*MCM9*) are implicated in homologous recombination and repair of DNA double-strand breaks. Female mice with *MCM8* and *MCM9* defects exhibit early oocyte loss or severe early proliferative defects in germ cells in testes [25]. The protein encoded by eukaryotic translation initiation factor 2B, subunit gamma (*EIF2B3*), is a subunit of the initiation factor *eIF2B*, which is associated with vanishing white matter (VWM) disease and leukodystrophy. Ovarian failure is frequently reported in women with VWM disease [26]. Mutations of the *EIF2B3* gene are very rarely found in patients with POF [27]. Mutations in prolyl endopeptidase like (*PREPL*) are associated with hypotonia-cystinuria syndrome (HCS), which is caused by deletions including *SLC3A1*. *PREPL* deficiency causes hypergonadotropic hypogonadism and HCS [28]. The *ERCC6*-*PGBD3* fusion protein and mutations in the ERCC excision repair 6 (*ERCC6*) gene are reported to cause POF and are among several proteins that participate in, or regulate, DNA repair [29]. The protein encoded by helicase for meiosis 1 (*HFM1*) is thought to be an ATP-dependent DNA helicase and is primarily expressed in germ-line cells, such as testis and ovary. Mutations in the HFM1 gene, which encodes a protein necessary for homologous recombination and synapsis during meiosis, are a cause of POF [30]. Spalt like transcription factor 4 (*SALL4*) plays important roles in maintaining the pluripotency of embryonic stem cells and regulating the embryonic development of many organisms. Similar to adult mice, *SALL4* is highly expressed in adult humans, with expression restricted to the testis and ovary [31]. A recent study of 50 patients with POF reported pathogenic mutations in *SALL4* (3/50 patients, p.Val181Met; p.Lys597Arg; p.Thr760Ile) and predicted that these variants had different effects on SALL4 activity [32]. The *SALL4* variant (NM_020436.3; c.3149T>C; p.Ile1050Thr) detected in our study was not one of the three previously reported mutations; however, it could be expected to also influence *SALL4* activity. Thyroglobulin (TG) is a precursor of the iodinated thyroid hormones, thyroxine (T4) and triiodothyronine (T3), and is synthesized predominantly by the thyroid gland. Mutations in *TG* are associated with the development of thyroid disorders, such as congenital hypothyroidism, thyroid cancer, and autoimmunity [33]. Previously, we reported that hypothyroidism due to *TG* mutations could lead to POF.[34, 35]. The *TG* variant (NM_003235.4; c.7364G>A; p.Arg2455His) identified in P3 may have resulted in hypothyroidism.

We also reported identical variants that did not correspond to known POF genes but were found in two or more individuals with POF (Additional file 1: Table S4). The *ADAMTS* protease family contributes to aspects of reproductive organ development and tissue morphogenesis, and dysregulation or functional alteration of *ADAMTS* proteins are associated with reproductive disorders, such as polycystic ovary syndrome and POF [36]. In chickens, expression of *ADAMTSL1* increases in the developing ovaries during gonadal differentiation [37]. *FER1L6* homologs in model organisms are involved in folliculogenesis and a copy number variant was reported in a patient with POF [38]. Solute carrier family 3 member 1 (*SLC3A1*) frameshift variant (NM_000341.3; c.1820delT; p.Leu607fs) has very low allele frequency in both the ExAC (MAF = 8.24E−06) and gnomAD (4.08E−06) databases. According to VarSome, this SLC3A1 variant has a strong association with Cystineuria. Cystineuria is a rare hereditary disease associated with kidney stones, which is primarily caused by mutations in two protein subunits (rBAT and b^0,+^AT) encoded by *SLC3A1* and *SLC7A9* [39].

POF is a highly heterogeneous disorder that can be caused by a variety of factors, suggesting that an unspecified number of variants can cause the condition. The increase of research use of NGS has led to the discovery of numerous disease-specific genes and variants in patients with POF; however, these results are rarely verified through sufficient downstream experiments and are not managed systematically, making in-depth study of the disease difficult. To establish the genetic pathogenesis of POF, efforts are needed to understand the complex genetic mechanisms involved; for example, using integrated databases and approaches to predict combinations of disease-associated variants.

## Conclusion

Sporadic POF is a highly heterogeneous condition with no common genetic cause. Hence, the scope for identifying genes and variants common to many individuals is limited. Here, we identified several variants and genes that may cause disease in 34 Korean patients with apparent isolated POF. We report novel candidate variants in *ADAMTSL1* and *FER1L6*, as well as alterations in genes previously reported to be associated with POF. In the study of disease, prediction of the risk associated with variants detected in patients by WES and in the candidate genes associated with the disease is a useful approach to find causative genetic variants. Our data confirm that WES is an efficient tool for studying the genetic causes of POF and can contribute to understanding of disease etiology. For more accurate identification of pathogenic variants, integration and systematic management of data collected from a number of WES studies of specific POF populations (e.g., family data and severe case populations) will be necessary.

## Supplementary information

**Additional file 1: Table S1. **List of POF/POI genes (131). List of candidate POF genes obtained from four public databases (DisGeNET, Monarch, MalaCards and NCBI:Gene) that collect genes and variants associated with human disease. **Table S2. **Primers used for confirming selected variants. **Table S3. **Genes with identical rare variants (MAF ≤ 0.01) detected in more than one individual.** Table S4. **Identical coding variants found in two or more individuals via whole-exome sequencing. VEST analysis generates values between 0 and 1, and scores ≥ 0.5 were classified as pathogenic variants and those < 0.5 as benign. CADD analysis generates a PHRED-like scaled value. Scores ≥ 20 were classified as pathogenic variants and those < 20 as benign. Variants satisfying both CADD ≥ 20 and VEST ≥ 0.5 were classified as pathogenic.

**Additional file 2: Figure S1.** Sanger validation. Sanger sequencing chromatogram showing nine candidate variants and one novel variant in ten patients with POF. Variant positions are indicated by (*). Images were extracted using SeqScanner v2 (Applied Biosystems, Foster City, CA, USA). Peaks, bases, and quality bars are shown. See SeqScanner v2 help for details.

## Data Availability

All data generated or analyzed in this study is included in published articles. Human genome version hg19/GRCh37 was used as a reference genome in this study and is available in the USCS Genome Browser website (https://hgdownload.soe.ucsc.edu/goldenPath/hg19/bigZips/hg19.fa.gz). VarSome (https://varsome.com/) was used for a comprehensive interpretation of variants. The sequence data of patients are available in the NCBI SRA under the accession number PRJNA668627. The sequence data of controls are available in the NCBI SRA under the accession number PRJNA601005. The controls used in this study are 10 control samples from PRJNA601005, and the NCBI SRA accession numbers are SAMN13840382, SAMN13840383, SAMN13840393, SAMN13840395, SAMN13840396, SAMN13840398, SAMN13840400, SAMN13840387, SAMN13840386 and SAMN13840385.

## Abbreviations

POF: Premature ovarian failure
NGS: Next generation sequencing
WES: Whole exome sequencing
SNV: Single nucleotide variant
InDel: Insertion/deletion variant
MAF: Minor allele frequency
FSH: Follicle stimulating hormone
gDNA: Genomic DNA
VWM: Vanishing white matter
HCS: Hypotonia–cystinuria syndrome

## References

[1] Ford ES, Li C, Zhao G, Tsai J (2011). Trends in obesity and abdominal obesity among adults in the United States from 1999–2008. Int J Obes (Lond).

[2] Shelling AN (2010). Premature ovarian failure. Reproduction.

[3] Welt CK (2008). Primary ovarian insufficiency: a more accurate term for premature ovarian failure. Clin Endocrinol (Oxf).

[4] Norling A, Hirschberg AL, Rodriguez-Wallberg KA, Iwarsson E, Wedell A, Barbaro M (2014). Identification of a duplication within the GDF9 gene and novel candidate genes for primary ovarian insufficiency (POI) by a customized high-resolution array comparative genomic hybridization platform. Hum Reprod.

[5] Qin Y, Jiao X, Simpson JL, Chen ZJ (2015). Genetics of primary ovarian insufficiency: new developments and opportunities. Hum Reprod Update.

[6] Tucker EJ, Grover SR, Bachelot A, Touraine P, Sinclair AH (2016). Premature ovarian insufficiency: new perspectives on genetic cause and phenotypic spectrum. Endocr Rev.

[7] Caburet S, Arboleda VA, Llano E, Overbeek PA, Barbero JL, Oka K, Harrison W, Vaiman D, Ben-Neriah Z, Garcia-Tunon I (2014). Mutant cohesin in premature ovarian failure. N Engl J Med.

[8] de Vries L, Behar DM, Smirin-Yosef P, Lagovsky I, Tzur S, Basel-Vanagaite L (2014). Exome sequencing reveals SYCE1 mutation associated with autosomal recessive primary ovarian insufficiency. J Clin Endocrinol Metab.

[9] AlAsiri S, Basit S, Wood-Trageser MA, Yatsenko SA, Jeffries EP, Surti U, Ketterer DM, Afzal S, Ramzan K, Faiyaz-Ul Haque M (2015). Exome sequencing reveals MCM8 mutation underlies ovarian failure and chromosomal instability. J Clin Invest.

[10] Li J, Zhao T, Zhang Y, Zhang K, Shi L, Chen Y, Wang X, Sun Z (2018). Performance evaluation of pathogenicity-computation methods for missense variants. Nucleic Acids Res.

[11] Carter H, Douville C, Stenson PD, Cooper DN, Karchin R (2013). Identifying Mendelian disease genes with the variant effect scoring tool. BMC Genomics.

[12] Kircher M, Witten DM, Jain P, O'Roak BJ, Cooper GM, Shendure J (2014). A general framework for estimating the relative pathogenicity of human genetic variants. Nat Genet.

[13] Nakagomi H, Mochizuki H, Inoue M, Hirotsu Y, Amemiya K, Sakamoto I, Nakagomi S, Kubota T, Omata M (2018). Combined annotation-dependent depletion score for BRCA1/2 variants in patients with breast and/or ovarian cancer. Cancer Sci.

[14] Nomura A, Tada H, Teramoto R, Konno T, Hodatsu A, Won HH, Kathiresan S, Ino H, Fujino N, Yamagishi M (2016). Whole exome sequencing combined with integrated variant annotation prediction identifies a causative myosin essential light chain variant in hypertrophic cardiomyopathy. J Cardiol.

[15] Lanktree MB, Haghighi A, Guiard E, Iliuta IA, Song X, Harris PC, Paterson AD, Pei Y (2018). Prevalence estimates of polycystic kidney and liver disease by population sequencing. J Am Soc Nephrol.

[16] Kopanos C, Tsiolkas V, Kouris A, Chapple CE, Albarca Aguilera M, Meyer R, Massouras A (2019). VarSome: the human genomic variant search engine. Bioinformatics.

[17] Lee Y, Kim C, Park Y, Pyun JA, Kwack K (2016). Next generation sequencing identifies abnormal Y chromosome and candidate causal variants in premature ovarian failure patients. Genomics.

[18] Li H, Durbin R (2010). Fast and accurate long-read alignment with Burrows–Wheeler transform. Bioinformatics.

[19] Li H, Handsaker B, Wysoker A, Fennell T, Ruan J, Homer N, Marth G, Abecasis G, Durbin R (2009). Genome project data processing S: the sequence alignment/map format and SAMtools. Bioinformatics.

[20] Tennessen JA, Bigham AW, O'Connor TD, Fu W, Kenny EE, Gravel S, McGee S, Do R, Liu X, Jun G (2012). Evolution and functional impact of rare coding variation from deep sequencing of human exomes. Science.

[21] Lek M, Karczewski KJ, Minikel EV, Samocha KE, Banks E, Fennell T, O'Donnell-Luria AH, Ware JS, Hill AJ, Cummings BB (2016). Analysis of protein-coding genetic variation in 60,706 humans. Nature.

[22] Sills ES, Brady AC, Omar AB, Walsh DJ, Salma U, Walsh AP (2010). IVF for premature ovarian failure: first reported births using oocytes donated from a twin sister. Reprod Biol Endocrinol.

[23] Jung KS, Hong KW, Jo HY, Choi J, Ban HJ, Cho SB, Chung M (2020). KRGDB: the large-scale variant database of 1722 Koreans based on whole genome sequencing. Database (Oxford).

[24] Untergasser A, Cutcutache I, Koressaar T, Ye J, Faircloth BC, Remm M, Rozen SG (2012). Primer3–new capabilities and interfaces. Nucleic Acids Res.

[25] Lutzmann M, Grey C, Traver S, Ganier O, Maya-Mendoza A, Ranisavljevic N, Bernex F, Nishiyama A, Montel N, Gavois E (2012). MCM8- and MCM9-deficient mice reveal gametogenesis defects and genome instability due to impaired homologous recombination. Mol Cell.

[26] Fogli A, Rodriguez D, Eymard-Pierre E, Bouhour F, Labauge P, Meaney BF, Zeesman S, Kaneski CR, Schiffmann R, Boespflug-Tanguy O (2003). Ovarian failure related to eukaryotic initiation factor 2B mutations. Am J Hum Genet.

[27] Fogli A, Gauthier-Barichard F, Schiffmann R, Vanderhoof VH, Bakalov VK, Nelson LM, Boespflug-Tanguy O (2004). Screening for known mutations in EIF2B genes in a large panel of patients with premature ovarian failure. BMC Womens Health.

[28] Regal L, Martensson E, Maystadt I, Voermans N, Lederer D, Burlina A, Juan Fita MJ, Hoogeboom AJM, Olsson Engman M, Hollemans T (2018). PREPL deficiency: delineation of the phenotype and development of a functional blood assay. Genet Med.

[29] Qin Y, Guo T, Li G, Tang TS, Zhao S, Jiao X, Gong J, Gao F, Guo C, Simpson JL (2015). CSB-PGBD3 mutations cause premature ovarian failure. PLoS Genet.

[30] Wang J, Zhang W, Jiang H, Wu BL (2014). Primary ovarian insufficiency C: mutations in HFM1 in recessive primary ovarian insufficiency. N Engl J Med.

[31] Kohlhase J, Heinrich M, Schubert L, Liebers M, Kispert A, Laccone F, Turnpenny P, Winter RM, Reardon W (2002). Okihiro syndrome is caused by SALL4 mutations. Hum Mol Genet.

[32] Wang Q, Li D, Cai B, Chen Q, Li C, Wu Y, Jin L, Wang X, Zhang X, Zhang F (2019). Whole-exome sequencing reveals SALL4 variants in premature ovarian insufficiency: an update on genotype-phenotype correlations. Hum Genet.

[33] Rubio IG, Medeiros-Neto G (2009). Mutations of the thyroglobulin gene and its relevance to thyroid disorders. Curr Opin Endocrinol Diabetes Obes.

[34] Pyun JA, Kang H, Kim J, Cha DH, Kwack K (2011). Thyroglobulin gene is associated with premature ovarian failure. Fertil Steril.

[35] Pyun JA, Kim S, Cha DH, Ko JJ, Kwack K (2012). Epistasis between the HSD17B4 and TG polymorphisms is associated with premature ovarian failure. Fertil Steril.

[36] Russell DL, Brown HM, Dunning KR (2015). ADAMTS proteases in fertility. Matrix Biol.

[37] Carre GA, Couty I, Hennequet-Antier C, Govoroun MS (2011). Gene expression profiling reveals new potential players of gonad differentiation in the chicken embryo. PLoS ONE.

[38] Ledig S, Ropke A, Wieacker P (2010). Copy number variants in premature ovarian failure and ovarian dysgenesis. Sex Dev.

[39] Rhodes HL, Yarram-Smith L, Rice SJ, Tabaksert A, Edwards N, Hartley A, Woodward MN, Smithson SL, Tomson C, Welsh GI (2015). Clinical and genetic analysis of patients with cystinuria in the United Kingdom. Clin J Am Soc Nephrol.

