# Supplementary material for: Identification of potential causal variants for premature ovarian failure by whole exome sequencing
Source: BMC Med Genomics. 2020 Oct 27;13:159. doi: 10.1186/s12920-020-00813-x (PMC7590468; doi:10.1186/s12920-020-00813-x)

**Figure 1S. Sanger validation.** Sanger sequencing chromatogram showing nine candidate variants and one novel variant in ten patients with POF. Variant positions are indicated by (*). Images were extracted using SeqScanner v2 (Applied Biosystems, Foster City, CA, USA). Peaks, bases, and quality bars are shown. See SeqScanner v2 help for details.

1) P3;TG;NM_003235.4;c.7364G>A;p.Arg2455His - forward/reverse


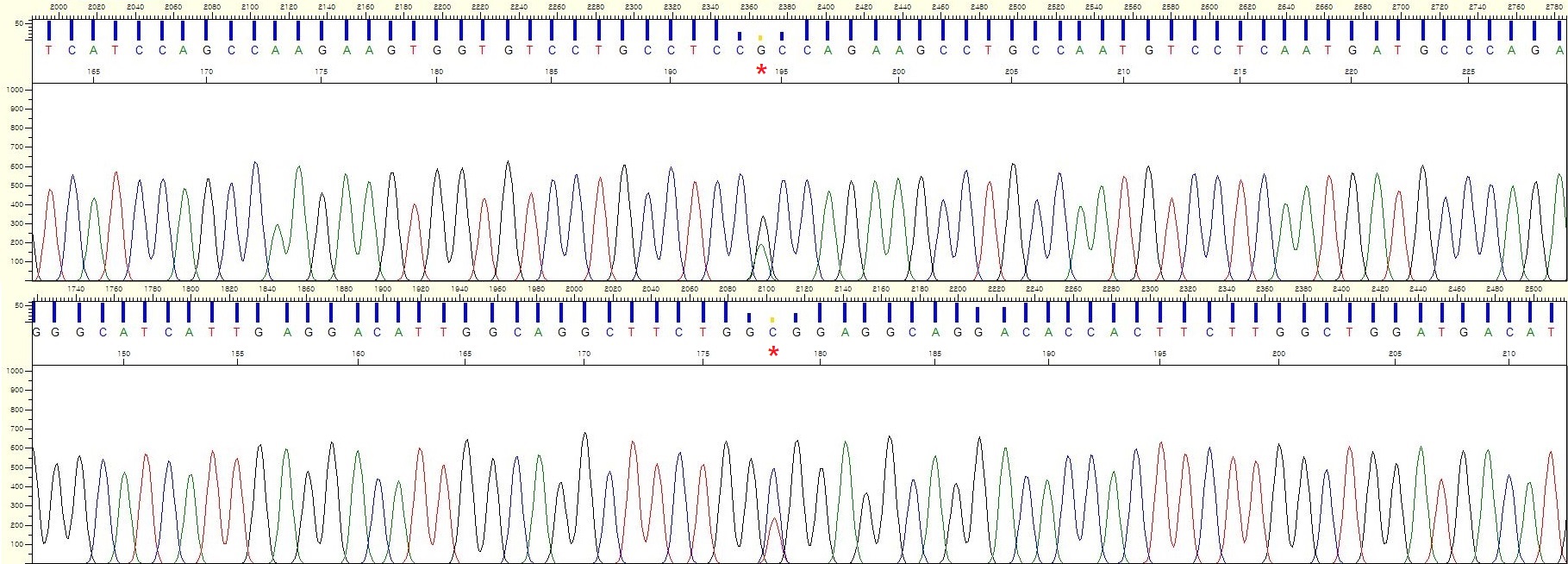


2) P5;ERCC6;NM_000124.3;c.2510G>A;p.Arg837His - forward/reverse


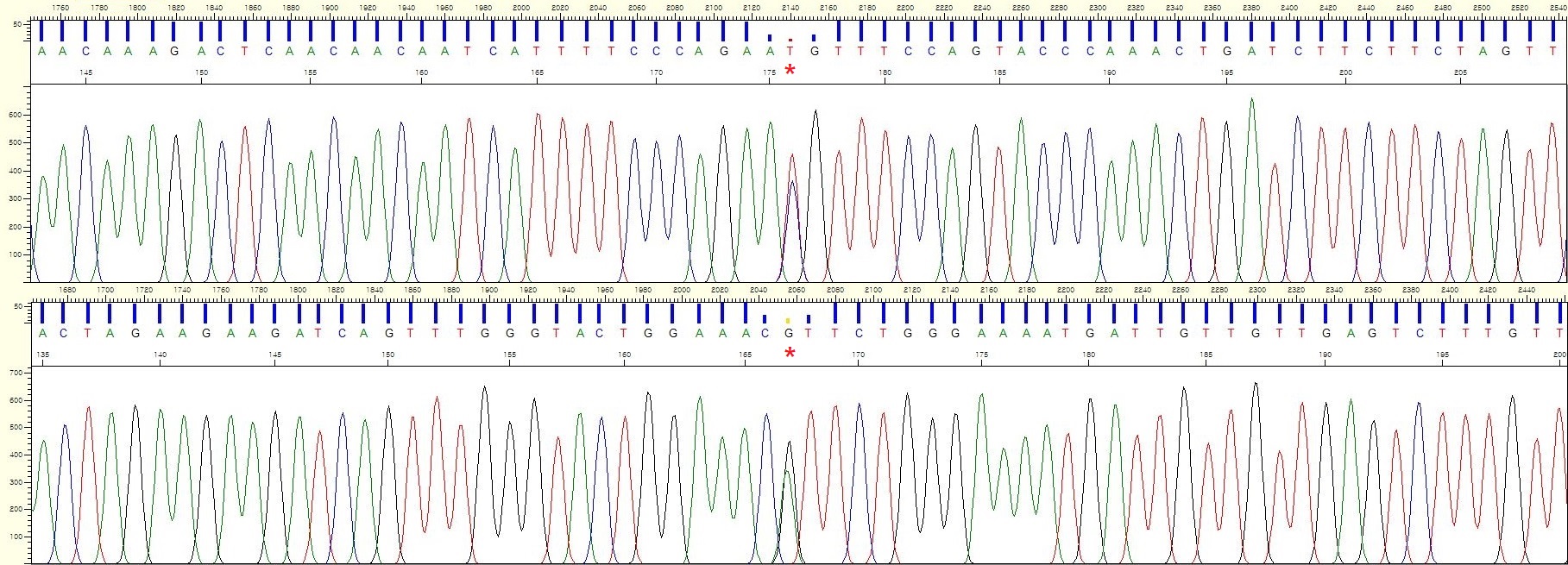


3) P14;MCM8;NM_001281521.1;c.1565C>T;p.Thr522Met - forward/reverse


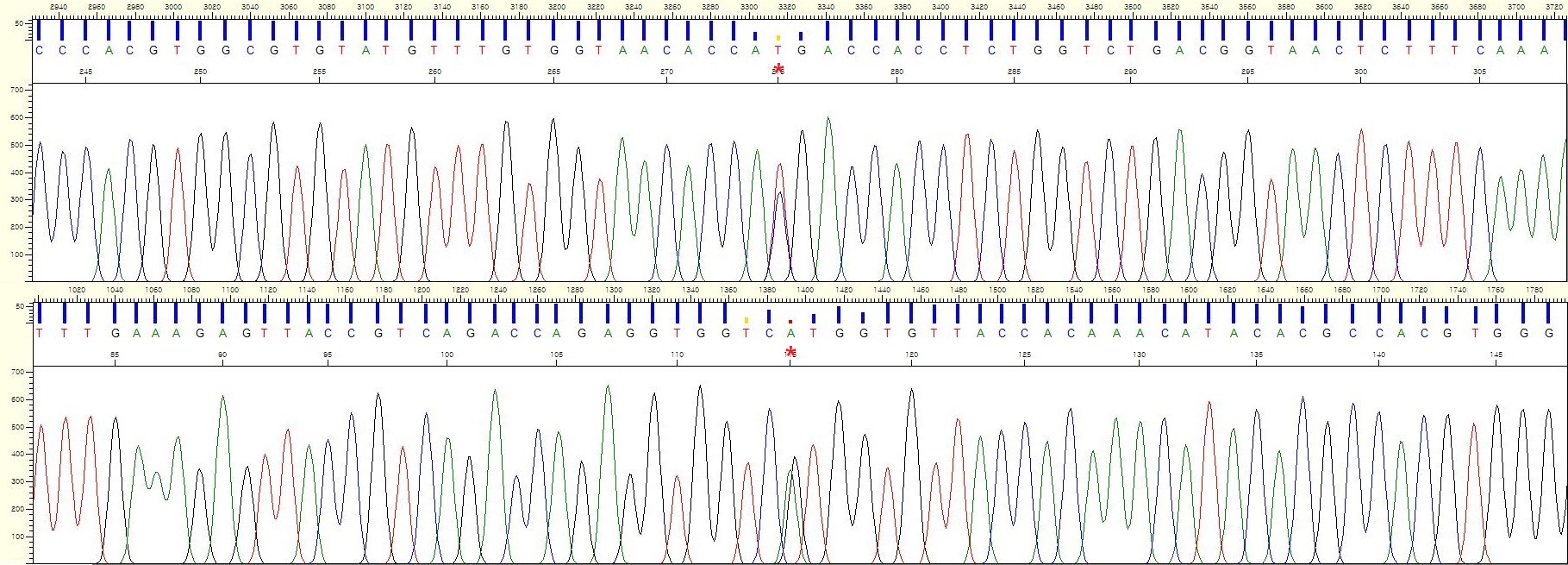


4) P28;MCM8;NM_001281521.1;c.1565C>T;p.Thr522Met - forward/reverse


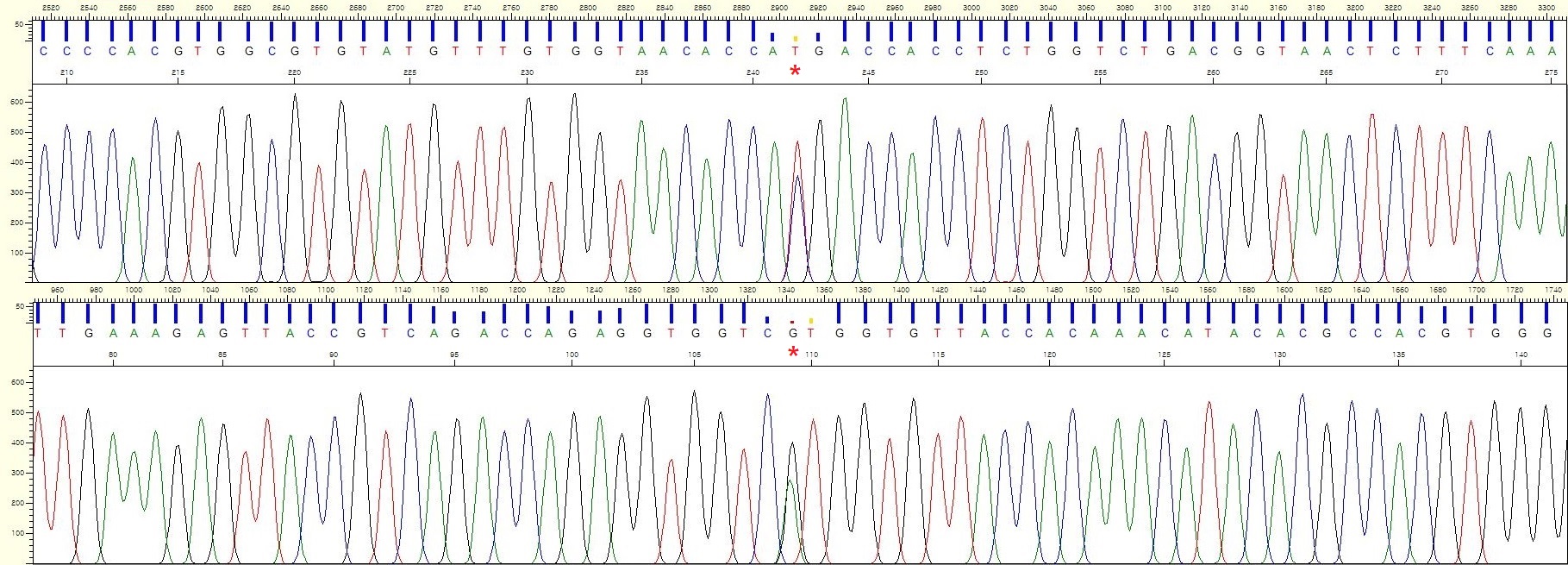


5) P15;EIF2B3;NM_020365.4;c.130G>A;p.Glu44Lys - forward/reverse


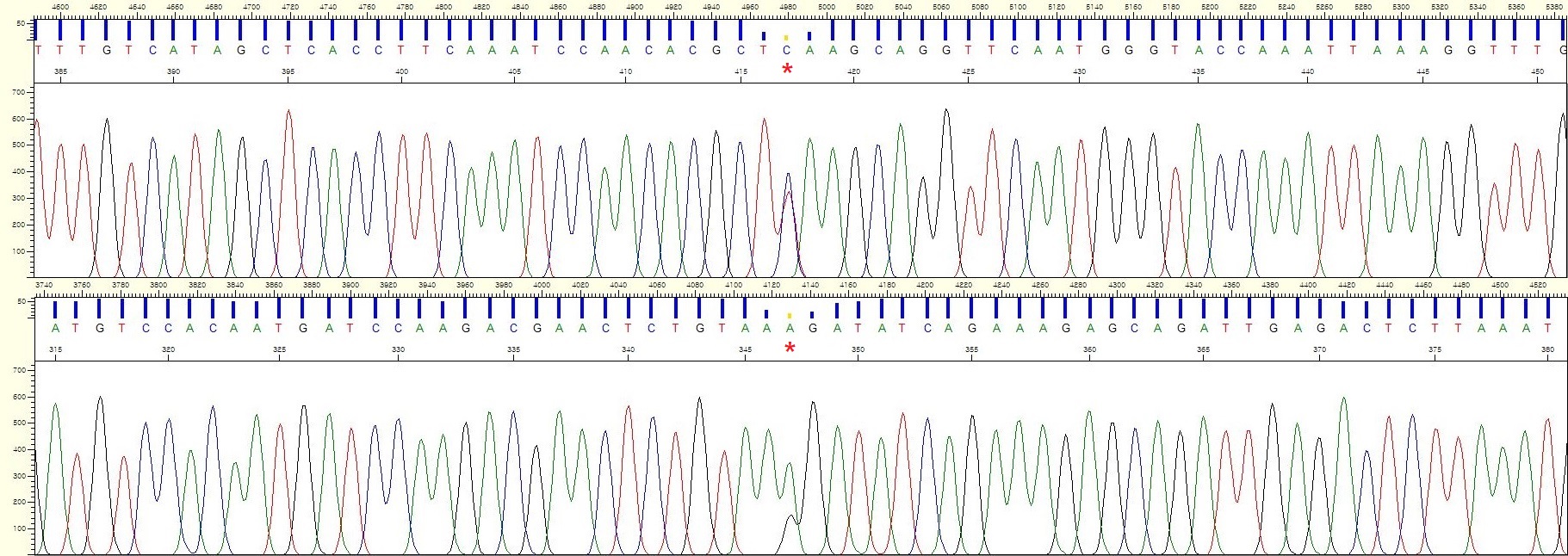


6) P15;PREPL;NM_001171603.1;c.1940G>A;p.Arg647Gln - forward/reverse


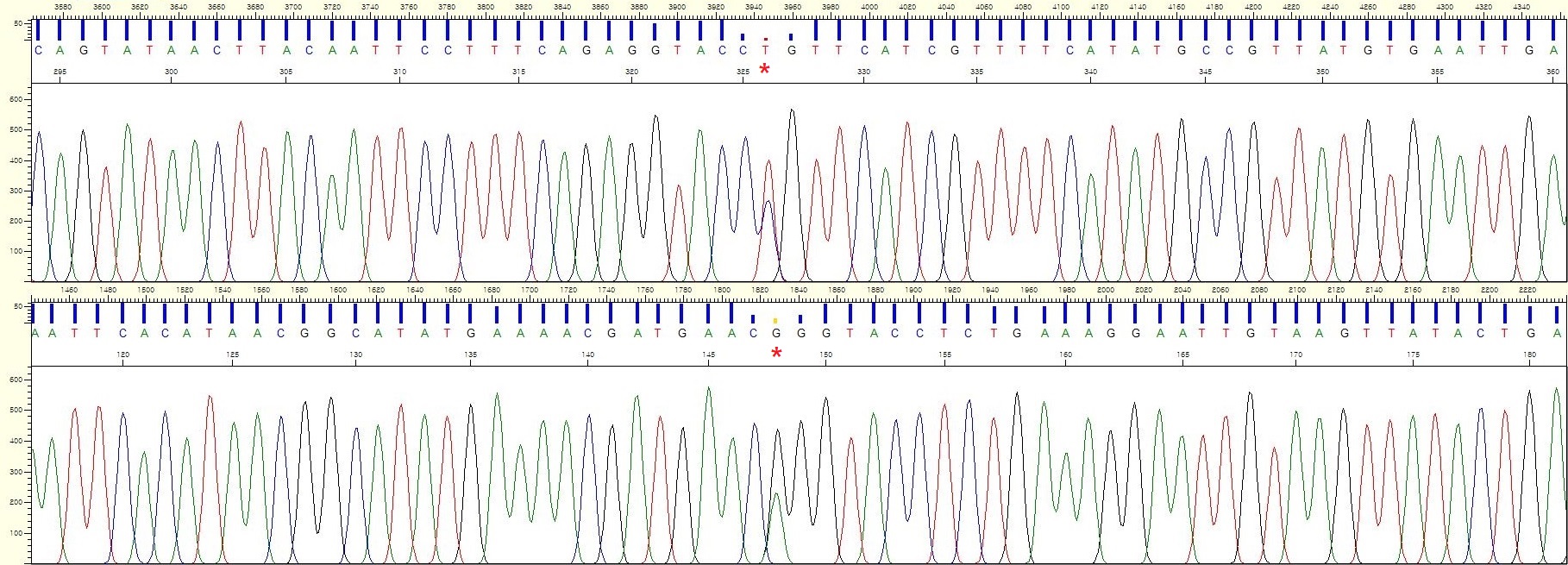


7) P23;HFM1;NM_001017975.4;c.3047A>G;p.Gln1016Arg - forward/reverse


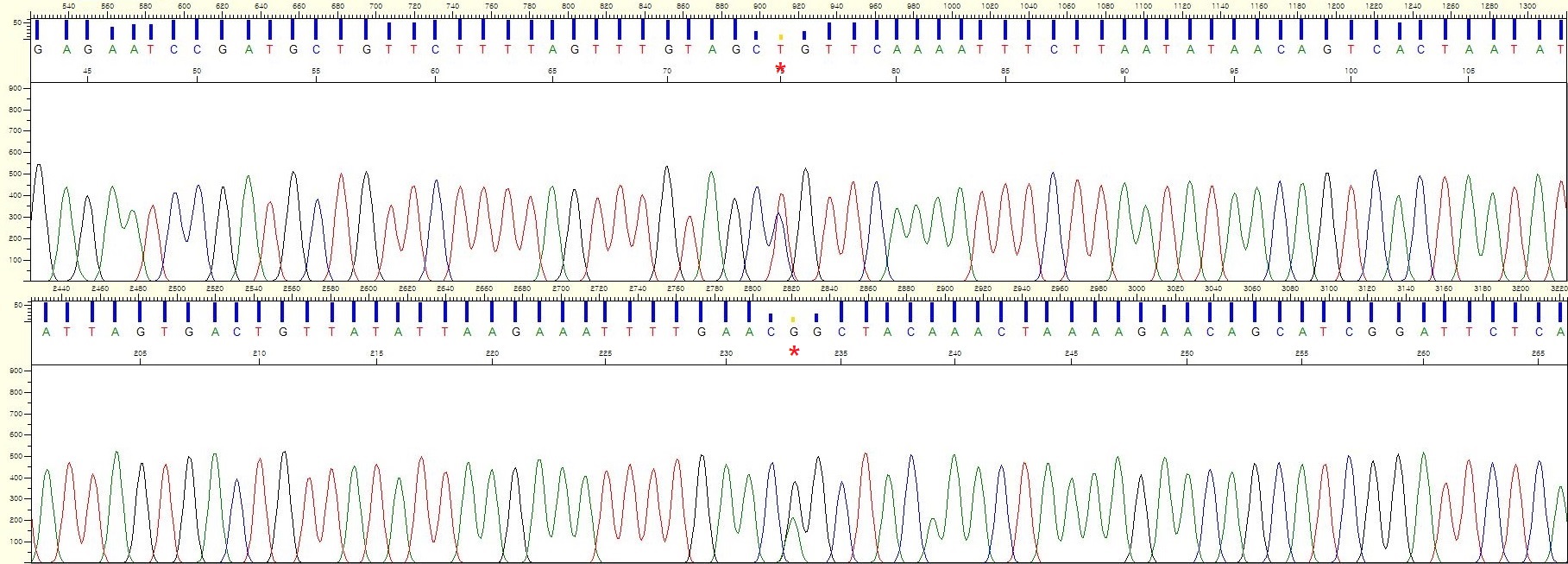


8) P23;SALL4;NM_020436.3;c.3149T>C;p.Ile1050Thr - forward/reverse


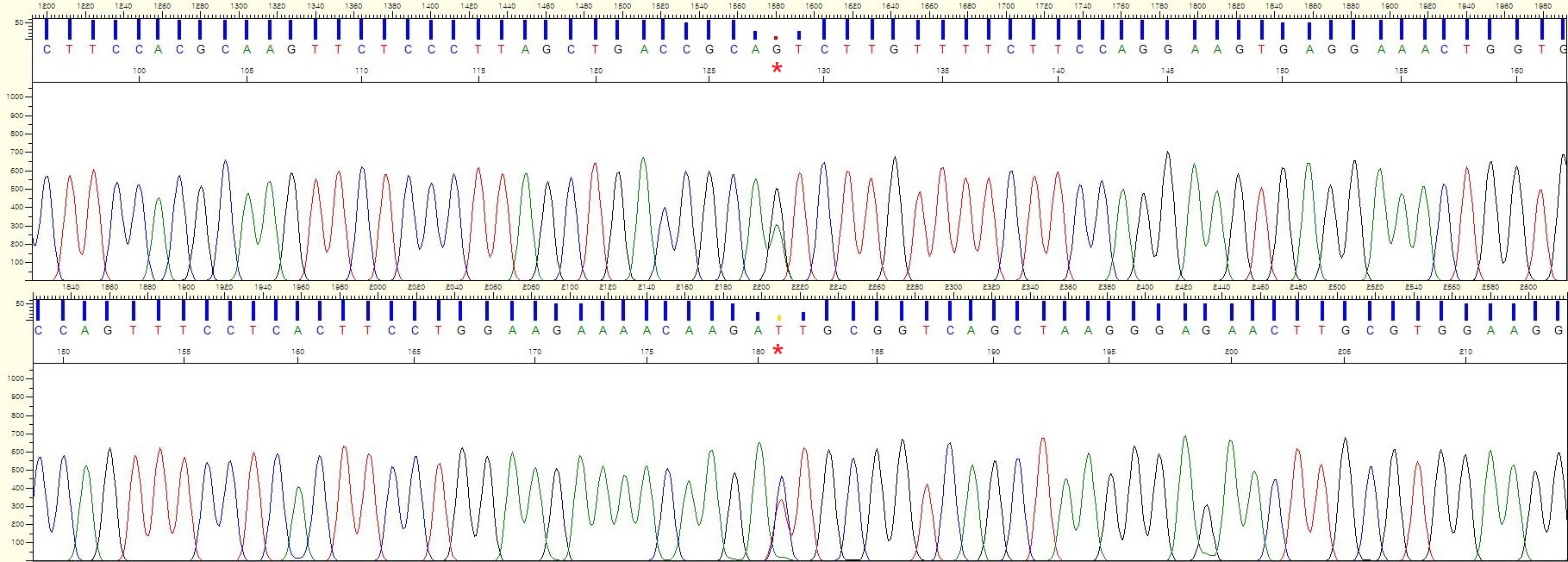


9) P26;MCM9;NM_017696.2;c.1330G>C;p.Val444Leu - forward/reverse


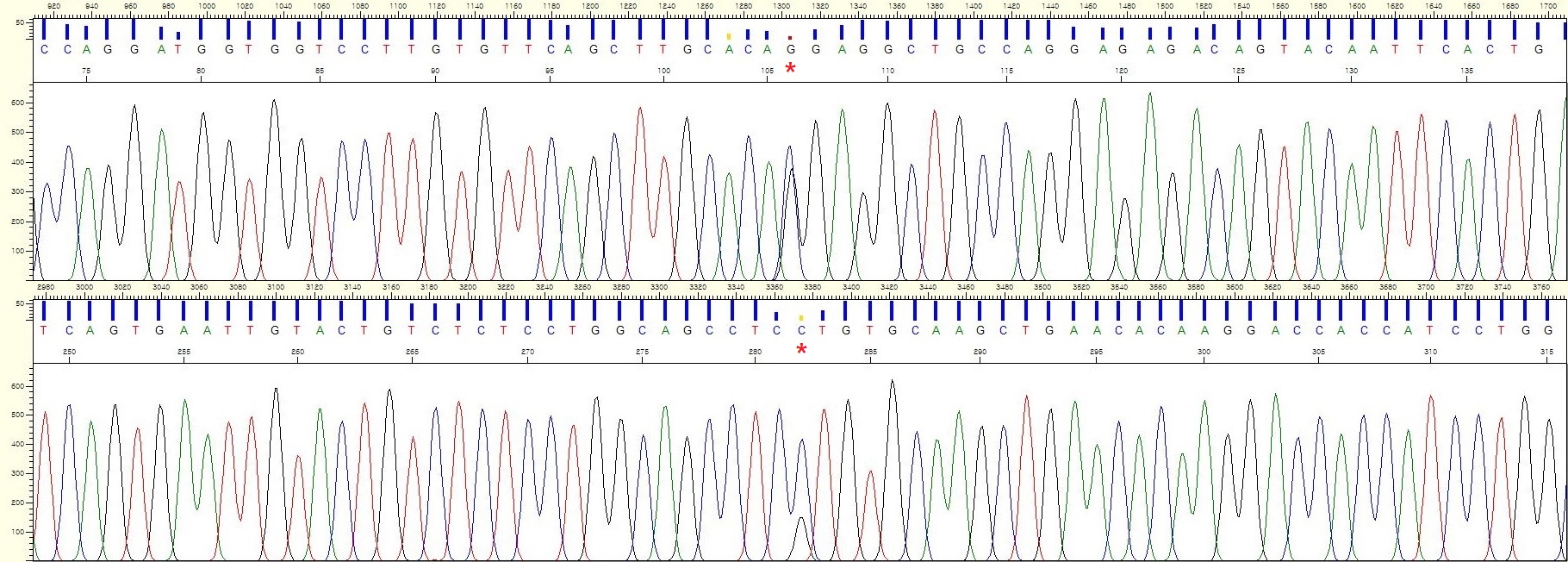


10) P32;MCM8;NM_001281521.1;c.839C>G;p.Ser280Cys - forward/reverse


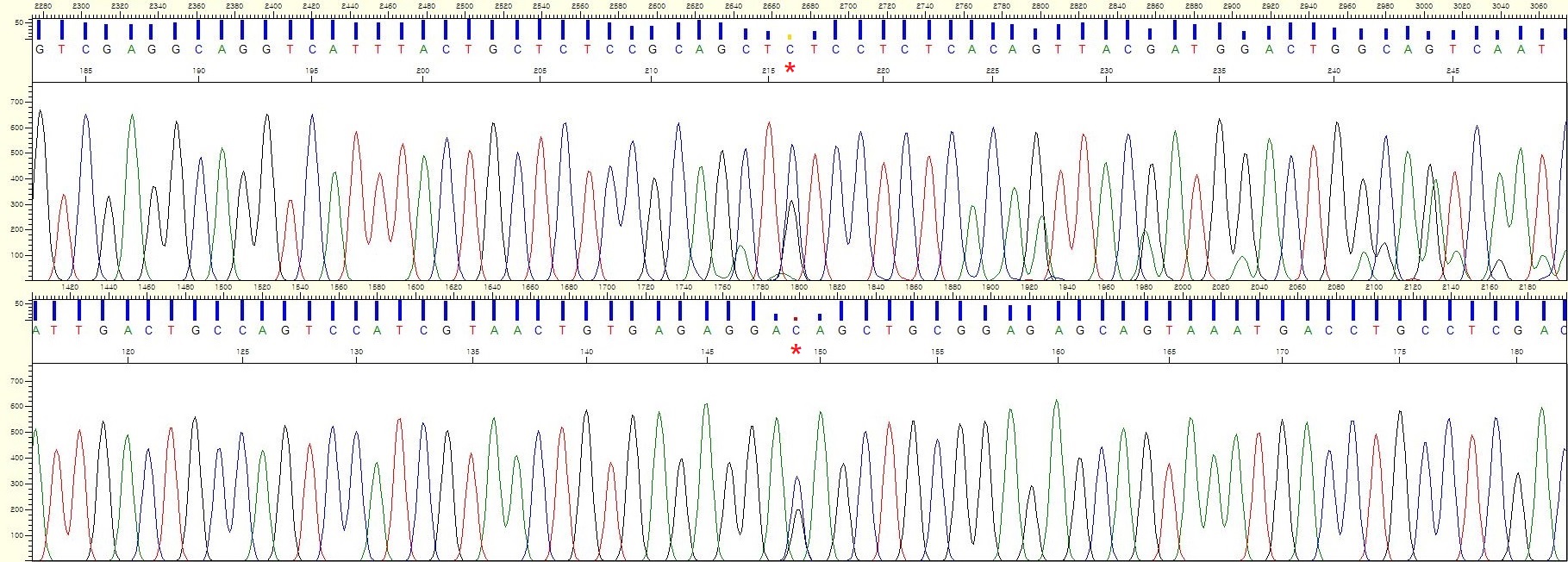

Supplement: Supplementary file 2 — Additional file 2: Figure S1. Sanger validation. Sanger sequencing chromatogram showing nine candidate variants and one novel variant in ten patients with POF. Variant positions are indicated by (*). Images were extracted using SeqScanner v2 (Applied Biosystems, Foster City, CA, USA). Peaks, bases, and quality bars are shown. See SeqScanner v2 help for details. [file 12920_2020_813_MOESM2_ESM.docx]
